# Editorial: Phenotypic transitions and endothelial dysfunction in cardiovascular diseases: mechanisms, therapeutic targets, and modulation

**DOI:** 10.3389/fphys.2025.1720883

**Published:** 2025-10-23

**Authors:** Laena Pernomian, Vanessa de Fátima Borges, Gerson Jhonatan Rodrigues, Cristina Espinosa-Diez

**Affiliations:** ^1^ Cardiovascular Translational Research Center, Department of Cell Biology and Anatomy, School of Medicine, University of South Carolina, Columbia, SC, United States; ^2^ Cedars Sinai Medical Center, Los Angeles, CA, United States; ^3^ Federal University of São Carlos, SãoCarlos, São Paulo, Brazil; ^4^ Center for Molecular Medicine and Genetics, School of Medicine, Wayne State University Detroit, Detroit, MI, United States

**Keywords:** endothelial dysfuction, vascular phenotypic transitions, cardiovascular diseases, therapeutic strategies, vascular damage

**FIGURE 1:**
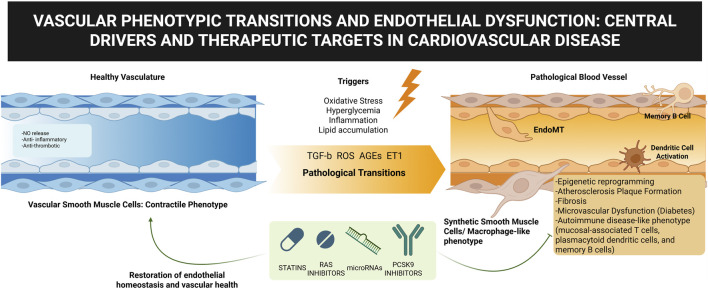
Vascular phenotypic transitions and endothelial dysfunction. Created with Biorender.com.

The vascular system is essential for organ function and tissue homeostasis, with endothelial cells regulating vascular tone, permeability, angiogenesis, and blood interactions. Disruption of this balance causes endothelial dysfunction, a central driver of cardiovascular and metabolic diseases. This is aggravated by maladaptive phenotypic transitions, particularly endothelial-to-mesenchymal transition (EndMT), which, though beneficial in repair, promotes fibrosis, remodeling, and plaque instability when dysregulated, contributing to atherosclerosis, diabetes, and hypertension (Kovacic et al., 2019). Understanding these transitions remains a challenge. This Research Topic explores the interplay between endothelial dysfunction and vascular cell phenotypes in disease progression (Figure 1).

Within the context of atherosclerosis, endothelial dysfunction represents an initiating and central event, as highlighted by Yang et al., who offer an integrative perspective bridging traditional pharmacotherapy with emerging novel therapeutic approaches. Their review emphasizes how endothelial and vascular cell phenotypic changes drive the disease process, through EndMT-derived fibroblast accumulation (Brokopp et al., 2011), extracellular matrix deposition and inflammation (Chen et al., 2015), and vascular smooth muscle cell (VSMC) switching (Chappell et al., 2016). Yang et al. illustrate how targeting maladaptive cell states through statins, renin-angiotensin system inhibitors, microRNAs, and reprogramming strategies may represent a paradigm shift for long-term vascular health.

A similar emphasis on endothelial dysfunction is evident in the context of diabetes. Here, Liu et al. expand on the multifactorial pathways, including oxidative stress, insulin resistance, and chronic hyperglycemia, that converge to impair endothelial cell function (Shah and Brownlee, 2016). By showing how these stressors reduce nitric oxide bioavailability, disrupt intercellular junctions, and trigger epigenetic modifications, their review underscores the endothelial cell as the primary target of diabetic vascular damage. Importantly, they also highlight how therapeutic strategies, including compounds from traditional Chinese medicine, may help preserve or restore endothelial function, positioning these cells as central targets for reducing cardiovascular risk in diabetes.

The contribution by Song et al. further extends these insights into the domain of plaque regression, challenging the notion of atherosclerosis as an irreversible condition. Their review emphasizes that regression is not simply the reversal of plaque buildup but rather a coordinated process involving lipid lowering, endothelial repair, and vascular cell reprogramming. Endothelial progenitor cells emerge as key agents of vascular repair, while VSMC phenotypic plasticity is highlighted as both a pathological driver and a therapeutic opportunity. By introducing conceptual parallels with oncology, such as targeting genomic instability, Song et al. propose innovative avenues for reshaping the therapeutic landscape of cardiovascular disease.

Beyond these vascular conditions, the clinical study by Jaatinen et al. explores ischemia with non-obstructive coronary arteries (INOCA), where endothelial dysfunction manifests in the coronary microvasculature. Their findings of altered immune responses point to a possible autoimmune contribution to microvascular dysfunction in INOCA. This aligns with prior evidence linking endothelial dysfunction and immune dysregulation in autoimmune diseases (Moschetti et al., 2022; Cecere et al., 2024), further reinforcing the idea that vascular pathology emerges from a convergence of endothelial injury, phenotypic transitions, and immune activation.

These studies illustrate the role of endothelial dysfunction and vascular cell phenotypic transitions in a wide spectrum of cardiovascular diseases. Endothelial cells, along with phenotypically plastic of vascular cells, constitute both key drivers of vascular injury and promising therapeutic targets. These findings highlight the importance of moving beyond symptom control to strategies that restore vascular homeostasis by correcting dysfunctional cellular states, thereby opening the door to transformative therapies for cardiovascular diseases.
